# Correlation between bacterial microbiome and *Legionella* species in water from public bath facilities by 16S rRNA gene amplicon sequencing

**DOI:** 10.1128/spectrum.03459-23

**Published:** 2024-02-16

**Authors:** Jun-ichi Kanatani, So Fujiyoshi, Junko Isobe, Keiko Kimata, Masanori Watahiki, Emi Maenishi, Shinji Izumiyama, Junko Amemura-Maekawa, Fumito Maruyama, Kazunori Oishi

**Affiliations:** 1 Department of Bacteriology, Toyama Institute of Health, Imizu, Toyama, Japan; 2 Section of Microbial Genomics and Ecology, Hiroshima University, Hiroshima, Japan; 3 Department of Parasitology, National Institute of Infectious Diseases, Toyama, Japan; 4 Department of Bacteriology, National Institute of Infectious Diseases, Toyama, Japan; University of Washington, Seattle, Washington, USA

**Keywords:** *Legionella*, microbiome, public bath

## Abstract

Public bath facilities are a major source of *Legionella* infections in Japan. In this study, we performed 16S rRNA gene amplicon sequencing to characterize the bacterial community in bath and shower water from public bath facilities, along with chemical parameters, and investigated the effect of the bacterial microbiome on the presence of *Legionella* species. Although no significant difference in bacterial community richness was observed between bath and shower water samples, there was a remarkable difference in the bacterial community structure between them. Distance-based redundancy analysis revealed that several factors (free residual chlorine, pH, and conductivity) were correlated with the bacterial community in bath water. The most abundant bacterial genera in the samples were *Pseudomonas* (13.7%) in bath water and *Phreatobacter* (13.6%) in shower water, as indicated by the taxonomic composition, and the dominant bacteria differed between these environmental samples. *Legionella pneumophila* was the most frequently detected *Legionella* species, with additional 15 other *Legionella* species detected in water samples. In *Legionella-*positive water samples, several unassigned and uncultured bacteria were enriched together. In addition, the co-occurrence network showed that *Legionella* was strongly interconnected with two uncultured bacteria. *Corynebacterium* and *Sphingomonas* negatively correlated with *Legionella* species. The present study reveals the ecology of *Legionella* species, especially their interactions with other bacteria that are poorly understood to date.

**IMPORTANCE:**

Public bath facilities are major sources of sporadic cases and outbreaks of *Legionella* infections. Recently, 16S rRNA gene amplicon sequencing has been used to analyze bacterial characteristics in various water samples from both artificial and natural environments, with a particular focus on *Legionella* bacterial species. However, the relationship between the bacterial community and *Legionella* species in the water from public bath facilities remains unclear. In terms of hygiene management, it is important to reduce the growth of *Legionella* species by disinfecting the water in public bath facilities. Our findings contribute to the establishment of appropriate hygiene management practices and provide a basis for understanding the potential health effects of using bath and shower water available in public bath facilities.

## INTRODUCTION

*Legionella* species are the causative agents of Legionnaires’ disease, a severe form of legionellosis, and potentially fatal pneumonia (1). Presently, 65 *Legionella* species have been identified (2), approximately half of which have been demonstrated to be pathogenic to humans, and the majority are considered potential human pathogens (3). *Legionella* species are ubiquitous in nature and have also been found in artificial environments such as cooling towers, baths, showers, and decorative fountains (4–7). Legionellosis can be acquired through inhalation of aerosolized water contaminated with *Legionella* species (8). Therefore, aquatic facilities are potential sources of sporadic cases and disease outbreaks. A previous study reported that public bath facilities are a major source of *Legionella* infection in Japan (9) and that several outbreaks have occurred in public bath facilities in Japan (10).

Japan is one of the world’s largest volcanic countries. As hot springs are widespread in Japan, many public bath facilities are developed around them. Many Japanese people visit public bath facilities and enjoy the prominent culture of bathing. In Toyama Prefecture, hot springs are widely distributed in most cities. The maintenance staff is responsible for either cooling hot spring water or warming non-hot spring water because presumably people prefer to bathe in water ranging in temperatures of about 37°C to 42 °C. Many public bath facilities have a circulation water system in which sanitization is carried out using a concentration of 0.4 mg/L free residual chlorine, in accordance with the guidelines for public bath facilities (11).

Recently, 16S rRNA gene amplicon sequencing has been used to analyze bacterial characteristics in various water samples from both artificial and natural environments, with a particular focus on *Legionella* species (12–16). These reports have revealed that the colonization of *Legionella* species in water environments is affected by multiple factors such as microbial community composition, chemical parameters, and the presence of protozoan hosts. In cooling-tower water, several taxa found were correlated with *Legionella* species, and continuous chlorine application reduced microbial diversity creating a non-permissive environment for *Legionella* species (14). In dental unit water, the presence of amoeba was positively associated with 10 bacterial species, including *Legionella* (15). These results provide fundamental data that contribute to the hygiene management of artificial environmental water systems. Although a bacterial microbiome that may coexist with *Legionella* species has been identified in bath water (16), the influence of water source and chemical parameters on the bacterial microbiome has not been analyzed. This study aimed to determine the relationship between the bacterial microbiome and *Legionella* species in water samples collected from public bath facilities. We performed 16S rRNA gene amplicon sequencing to characterize the bacterial community in the water environments of public bath facilities, along with their chemical parameters, and investigated their interactions with *Legionella* species.

## RESULTS

### Sequencing analysis

The 16S rRNA gene amplicon sequencing was performed on water samples collected from bath water (*N* = 87) and shower water (*N* = 51) at public bath facilities (Table S1). Two samples (one from the bath and one from the shower) were removed from the analysis because of the low number of reads obtained (43 and 86 reads, respectively). The median number of reads after quality filtering, denoising, merging, and removal of chimeric sequences was 95,270 (range: 9,892–202,962) from bath water and 100,930 (range: 20,774–187,709) from shower water. The sequences from all samples were normalized to the sample containing the lowest number of reads (9,892 reads) and were assigned to 28,286 amplicon sequence variants (ASVs).

### Comparison of the bacterial community structure

**Fig 1:**
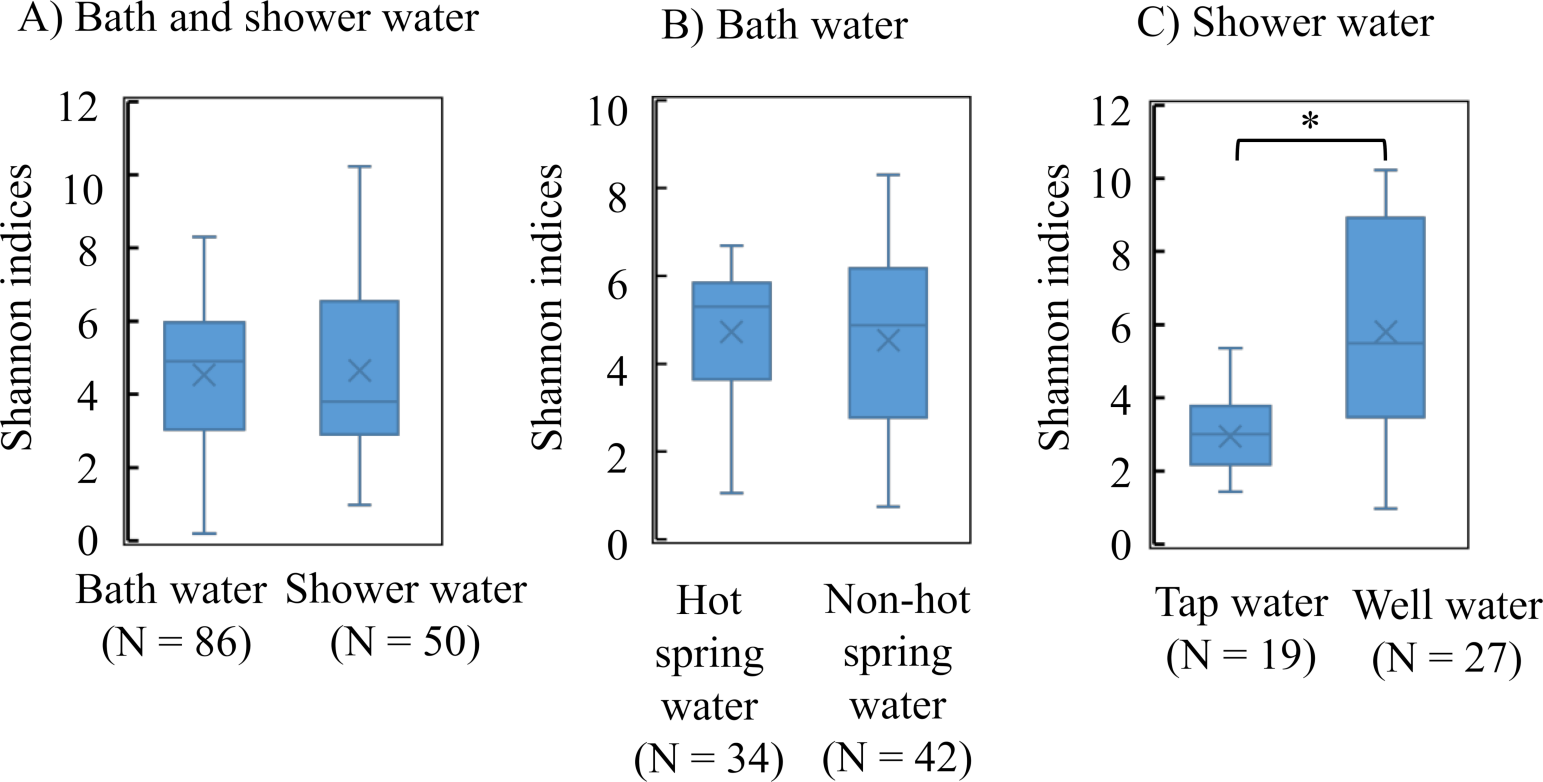
Comparisons of alpha diversities in bath and shower water (**A**), bath water from hot spring water and non-hot spring water (**B**), and shower water from tap and well water (**C**). Box plots represent (from top to bottom) maximum, upper-quartile, median, lower-quartile, and minimum values. Cross marks indicate the mean for each sample group.

No difference was found in the Shannon indices between bath water and shower water (Fig. 1A). Regarding the water source, no differences were found in the Shannon indices between hot spring water and non-hot spring water supplied to baths (Fig. 1B). In contrast, significant differences were observed in the Shannon indices between tap water and well water supplied to showers (Fig. 1C). The Japanese guidelines indicate that the free residual chlorine concentration in bath water should be kept at 0.4 mg/L (11). In bath water, the Shannon indices in bath water with free residual chlorine concentrations <0.4 mg/L were significantly lower than those with free residual chlorine concentrations ≥0.4 mg/L (Fig. S1A).

**Fig 2:**
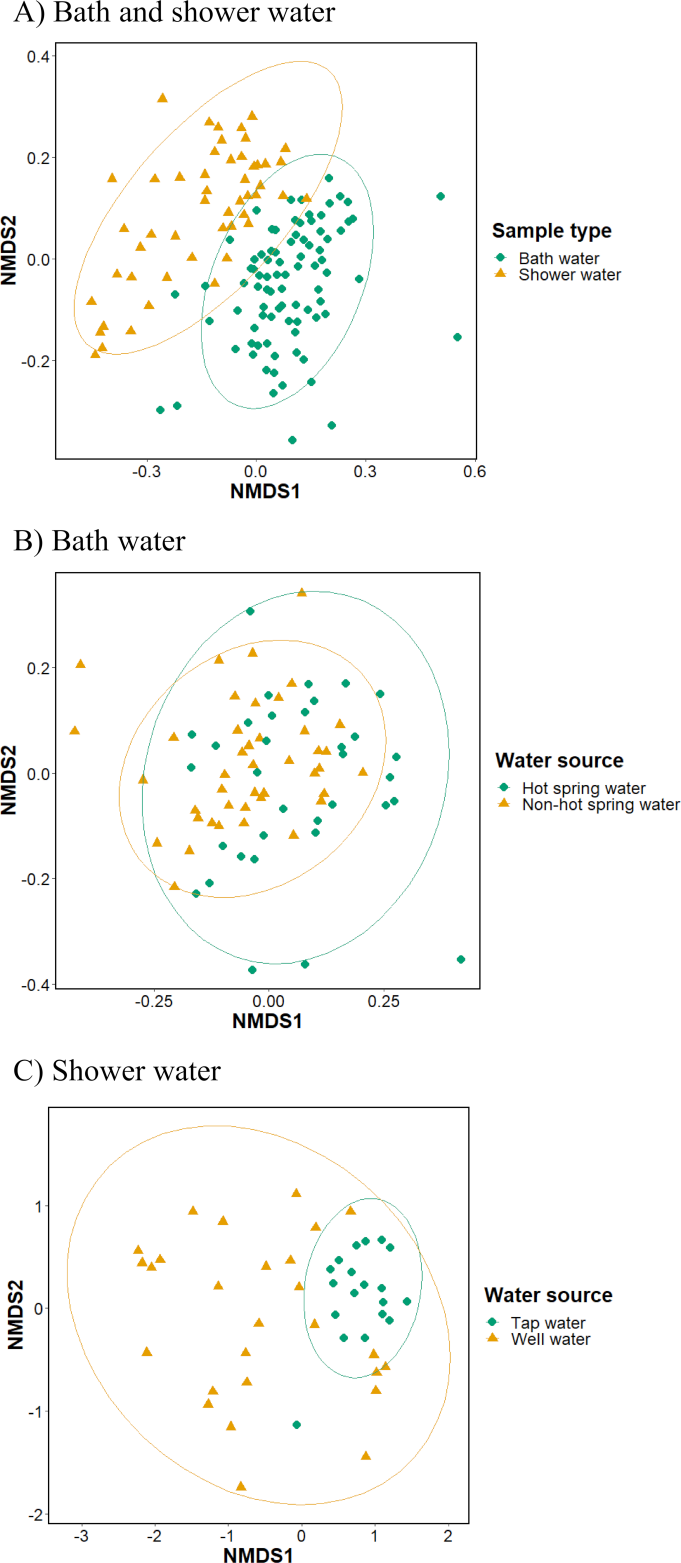
Comparisons of beta diversities in bath and shower water (**A**), bath water from hot spring water and non-hot spring water (**B**), and shower water from tap and well water (**C**). An NMDS plot showed the dissimilarity of the bacterial community. Ellipses represent 95% confidence intervals.

A nonmetric multidimensional scaling (NMDS) plot showed that bath water samples were distinct from shower water samples (Fig. 2A). However, the hot spring water was not distinctly different from the non-hot spring water (Fig. 2B). The analysis of shower water showed limited diversity in tap water samples but wide diversity in well water samples (Fig. 2C).

### Characteristics of the bacterial community composition

The taxonomic classification assigned 227 bacterial classes or candidate class-level lineages to each sample. The top three classes were Gammaproteobacteria (32.3%), Alphaproteobacteria (20.5%), and Bacilli (11.8%) in the bath water samples and Alphaproteobacteria (55.3%), Gammaproteobacteria (15.5%), and Vampirivibrionia (4.5%) in the shower water samples. Vampirivibrionia (17–19) and Omnitrophia (20) are not validly published classes in the List of Prokaryotic names with Standing in Nomenclature (LPSN) but have been found in the microbiota such as rats, dogs, and water. Alphaproteobacteria were especially abundant in shower water samples derived from tap water (82.9%) (Fig. S2).

At the genus level, 1,563 and 1,186 bacterial genera or candidate genus-level lineages were detected in the samples collected from bath and shower water, respectively. The three most abundant bacterial genera or candidate genus-level lineages were *Pseudomonas* (13.7%), *Staphylococcus* (8.8%), and *Candidatus-Obscuribacter* (6.2%) in bath water samples and *Phreatobacter* (13.6%), *Sphingomonas* (9.6%), and DSSF69 (family Sphingomonadaceae, 7.7%) in shower water samples (Table 1). DSSF69 and SM2D12 (order Rickettsiales) are undescribed genera in the LPSN. These genera include many sequences derived from uncultured bacteria and metagenomic samples in the Silva database. The three unassigned genera and uncultured bacteria are listed in Table 1. Reads from *Legionella* species were detected in 81/136 of total samples (63%): 54/86 samples (63%) were collected from bath water (mean proportion of reads, 0.6%), and 27/50 samples (54%) were collected from shower water (mean proportion of reads, 0.2%).

**TABLE 1:** Taxonomic composition of the 10 most abundant genera or candidate genus-level lineages^*a*^

Bath	Mean % (range)	No. (%) of detected samples	Shower	Mean % (range)	No. (%) of detected samples
*Pseudomonas*	13.7 (0–94.1)	84 (98)	*Phreatobacter*	13.6 (0–95.2)	32 (64)
*Staphylococcus*	8.8 (0–66.8)	74 (86)	*Sphingomonas*	9.6 (0–72.4)	49 (98)
*Candidatus-Obscuribacter*	6.2 (0–68.6)	67 (78)	DSSF69 (family Sphingomonadaceae)	7.7 (0–78.5)	37 (74)
Not assigned no. 1 (family Hyphomonadaceae)	5.3 (0–95.4)	47 (55)	*Novosphingobium*	6.2 (0–44.1)	46 (92)
Not assigned no. 2 (phylum Cyanobacteria)	3.6 (0–27.6)	75 (87)	*Mycobacterium*	3.6 (0–24.4)	42 (84)
*Mycobacterium*	3.4 (0–93.7)	78 (91)	*Methylobacterium-Methylorubrum*	3.2 (0–23)	44 (88)
*Methylobacterium-Methylorubrum*	3.4 (0–46.7)	73 (85)	*Bradyrhizobium*	3.1 (0–41.4)	48 (96)
Uncultured bacterium (family Hydrogenophilaceae)	3.2 (0–83.5)	21 (24)	Not assigned no. 3 (phylum Cyanobacteria)	2.8 (0–87.5)	46 (92)
*Sphingomonas*	2.0 (0–52.6)	75 (87)	*Blastomonas*	2.7 (0–54)	33 (66)
*Cutibacterium*	2.0 (0–81.4)	62 (72)	SM2D12 (order Rickettsiales)	2.2 (0–37.6)	38 (76)

^*a*^ DSSF69 and SM2D12 are undescribed genera in the LPSN. The higher taxa validly published in the LPSN are shown in parentheses.

### Distribution of *Legionella* species at the species level

In this study, *Legionella pneumophila* was the most frequently detected species; 32/86 samples (37%) were collected from bath water, and 9/50 samples (18%) were collected from shower water (Table 2). The mean proportion of the reads in the *L. pneumophila*-DNA positive samples was 0.23% (range: 0.01%–2.27%). Furthermore, 15 non-*L. pneumophila* species were detected. Of these, 10 species have been reported to be associated with human infection (21–30). At the species level, 2 or more species were detected in 25 bath water samples and 6 shower water samples (Table S2). In the sample with the largest number of *Legionella* species detected, the DNA of six *Legionella* species was found in the water from one of the baths.

**TABLE 2:** Distribution of *Legionella* species at the species level

Species	No. of *Legionella* DNA-detected samples:		Mean % (range) of *Legionella* DNA-positive samples	Human infection
	Bath	Shower		
*Legionella anisa*	1	2	0.06 (0.03–0.08)	Yes (21)
*Legionella donaldsonii* ^*a*^	0	1	0.01	Unknown
*Legionella drancourtii*	0	1	0.01	Unknown
*Legionella geestiana*	2	0	3.14 (0.02–6.27)	Unknown
*Legionella jordanis*	2	0	1.74 (0.02–3.46)	Yes (22)
*Legionella londiniensis*	3	0	0.76 (0.01–2.22)	Yes (23)
*Legionella maceachernii*	23	3	0.14 (0.01–0.69)	Yes (24)
*Legionella micdadei*	8	0	1.55 (0.03–5.74)	Yes (25)
*Legionella nautarum*	2	0	0.22 (0.01–0.43)	Unknown
*Legionella norrlandica*	1	0	1.55	Unknown
*Legionella oakridgensis*	2	0	0.02 (0.02–0.02)	Yes (26)
*Legionella pneumophila*	32	9	0.23 (0.01–2.27)	Yes (27)
*Legionella quinlivanii*	2	0	0.04 (0.01–0.06)	Yes (28)
*Legionella rubrilucens*	3	1	0.03 (0.01–0.06)	Yes (29)
*Legionella thermalis*	2	0	0.12 (0.04–0.19)	Unknown
*Legionella waltersii*	0	6	0.08 (0.01–0.22)	Yes (30)

^*a*^ Not validly published species in the LPSN.

In this study, plate culture method was used for all the water samples. The results also claimed *L. pneumophila* as the most frequently detected species with 11/86 samples (13%) collected from bath water and 7/50 samples (14%) collected from shower water (Table S1). Sequence-based typing of *L. pneumophila* serogroup 1 isolates revealed five sequence types (STs) detected in isolates from patients with legionellosis in Japan (ST129, ST138, ST328, ST502, and ST505) (31). Only three non-*L*. *pneumophila* species were isolated: *Legionella erythra* (one sample), *Legionella micdadei* (one sample), and *Legionella rubrilucens* (one sample). Among these, *L. micdadei* and *L. rubrilucens* are reportedly associated with human infections.

### Correlation between bacterial microbiome and *Legionella* species

**Fig 3:**
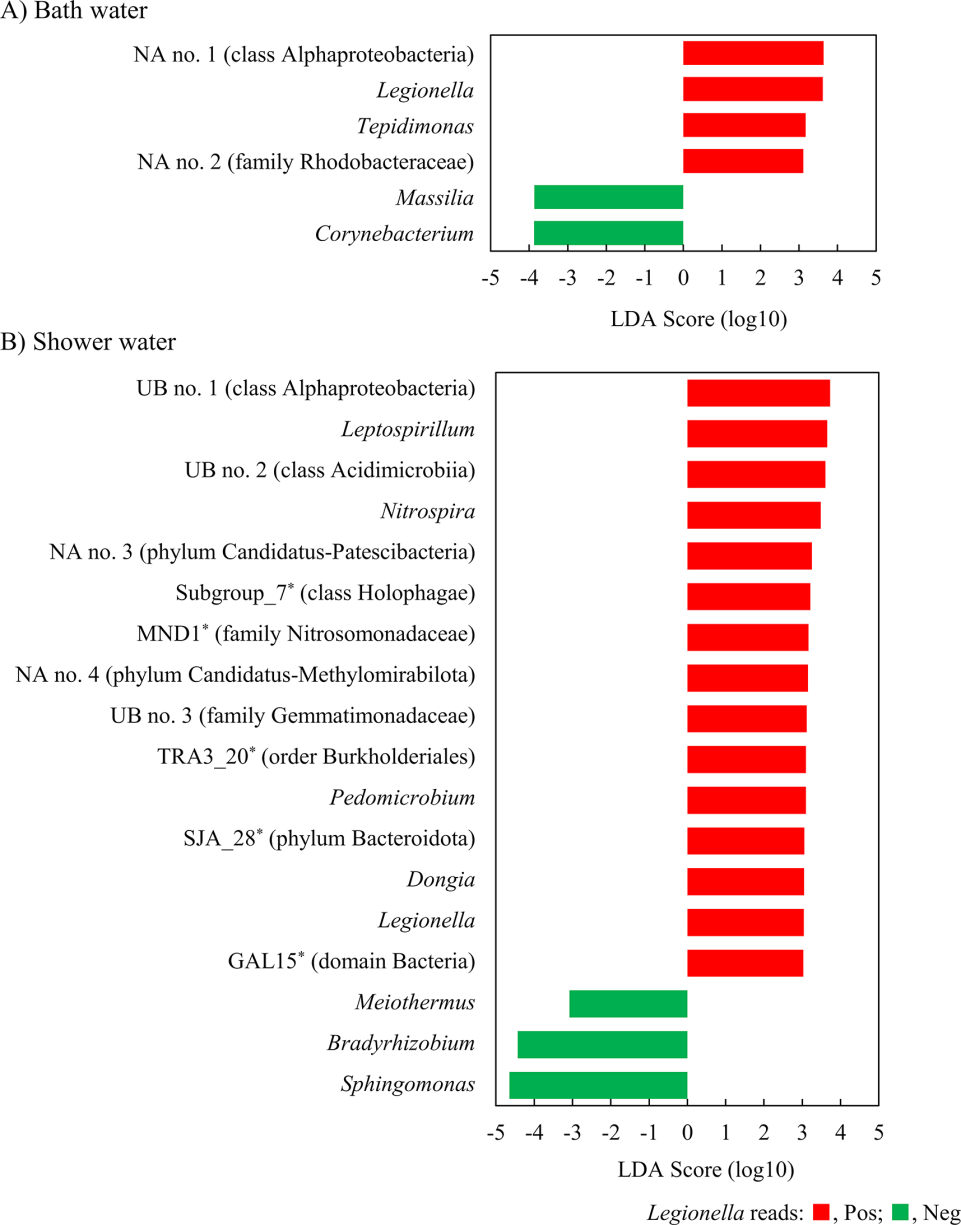
LEfSe identified the significantly different taxa between *Legionella*-read positive and negative samples in bath water (A) and shower water (B). The logarithmic LDA scores indicate the degree of consistent differences in relative abundance between the *Legionella*-read positive and negative samples. The LDA score is 3.0, with a *P* value less than 0.05 (pairwise Wilcoxon test). Asterisks indicate the undescribed genera in the LPSN. The higher taxa validly published in the LPSN are shown in parentheses. NA, unassigned genera; UB, uncultured bacterium.

Linear discriminant analysis (LDA) effect size (LEfSe) analysis revealed that four genera were enriched in *Legionella*-positive samples and two genera were enriched in *Legionella*-negative samples, respectively, from all bath water samples (Fig. 3). In shower water samples, 15 genera were enriched in *Legionella*-positive samples and three genera were enriched in *Legionella*-negative samples. Specifically, in bath water, *Tepidimonas* and *Corynebacterium* were enriched in *Legionella*-positive and -negative samples, respectively. In shower water, *Leptospirillum* and *Nitrospira* were enriched in *Legionella*-positive samples, and *Sphingomonas* was enriched in *Legionella*-negative samples. In addition, among the *Legionella-*positive samples, four unassigned genera and three uncultured bacteria were enriched in bath and shower water samples, respectively. Furthermore, in shower water, five detected genera (Subgroup_7, MND1, TRA3_20, SJA_28, and GAL15) are undescribed in the LPSN. These genera included many sequences derived from uncultured bacteria in the Silva database.

**Fig 4:**
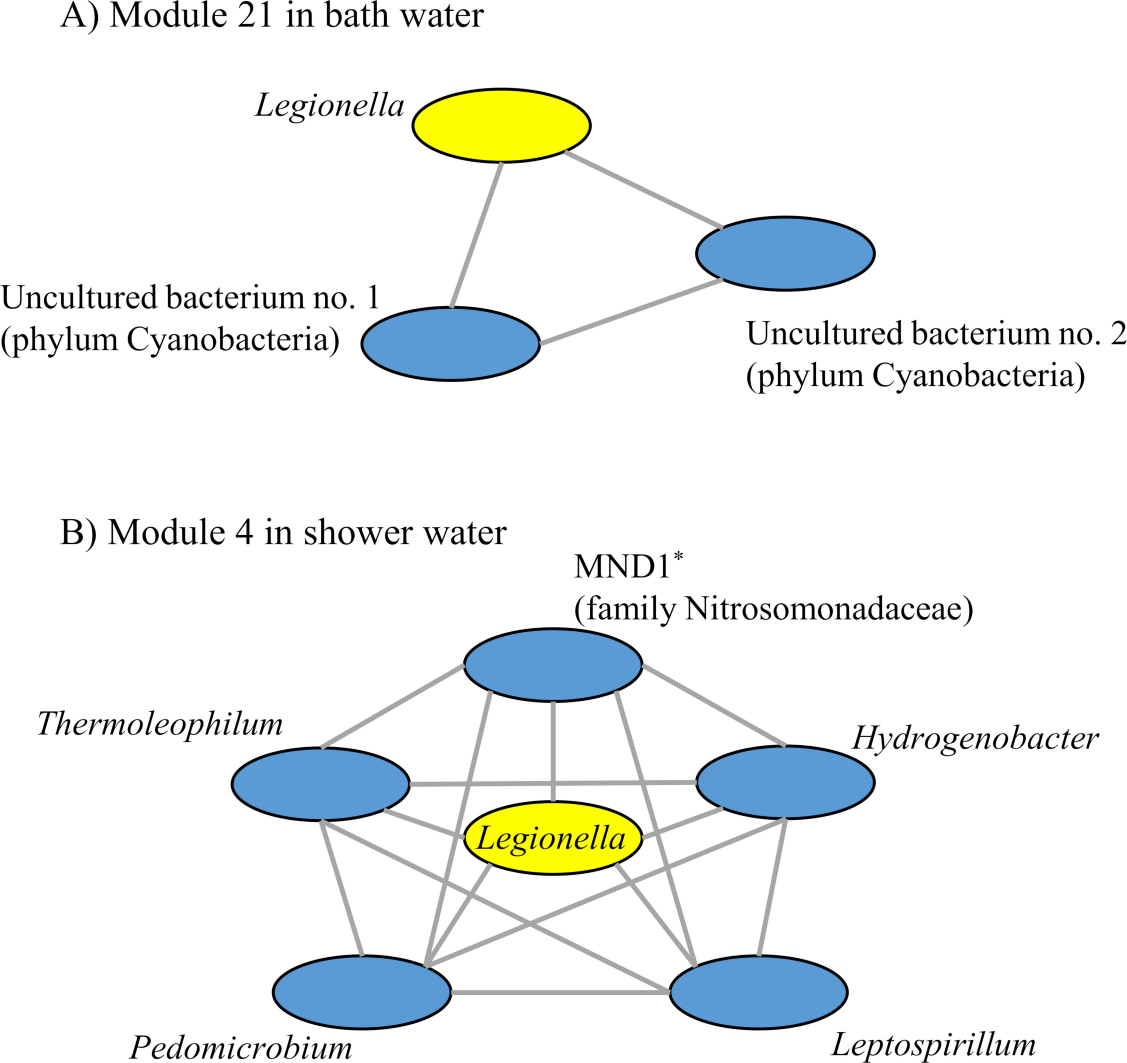
Correlation network analysis of bacterial microbiome in bath and shower water. Modules are groups in which the organisms are strongly correlated with each other and connected with an edge. Each node corresponds to ASVs, and edges in the network represent the positive correlations between the different nodes. Modules including taxa of *Legionella* in bath water (**A**) and shower water (**B**) are shown. Asterisks indicate the undescribed taxa in the LPSN. The higher taxa validly published in the LPSN are shown in parentheses.

Next, a sparse co-occurrence network investigation for compositional data (SCNIC) analysis revealed that a total of 49 and 74 modules were detected, including 138 and 249 ASVs in the bath and shower water samples, respectively (Table S3 and S4). Among these, two modules (module 21 in bath water and module 4 in shower water) contained ASVs from *Legionella* (Fig. 4). They showed that module 21 included ASVs from two uncultured bacterial species (phylum Cyanobacteria). In module 4, *Leptospirillum*, MND1, and *Pedomicrobium* were also detected in *Legionella*-positive samples derived from shower water using LEfSe analysis.

### Correlation between environmental variables and dissimilarity of bacterial community

**Fig 5:**
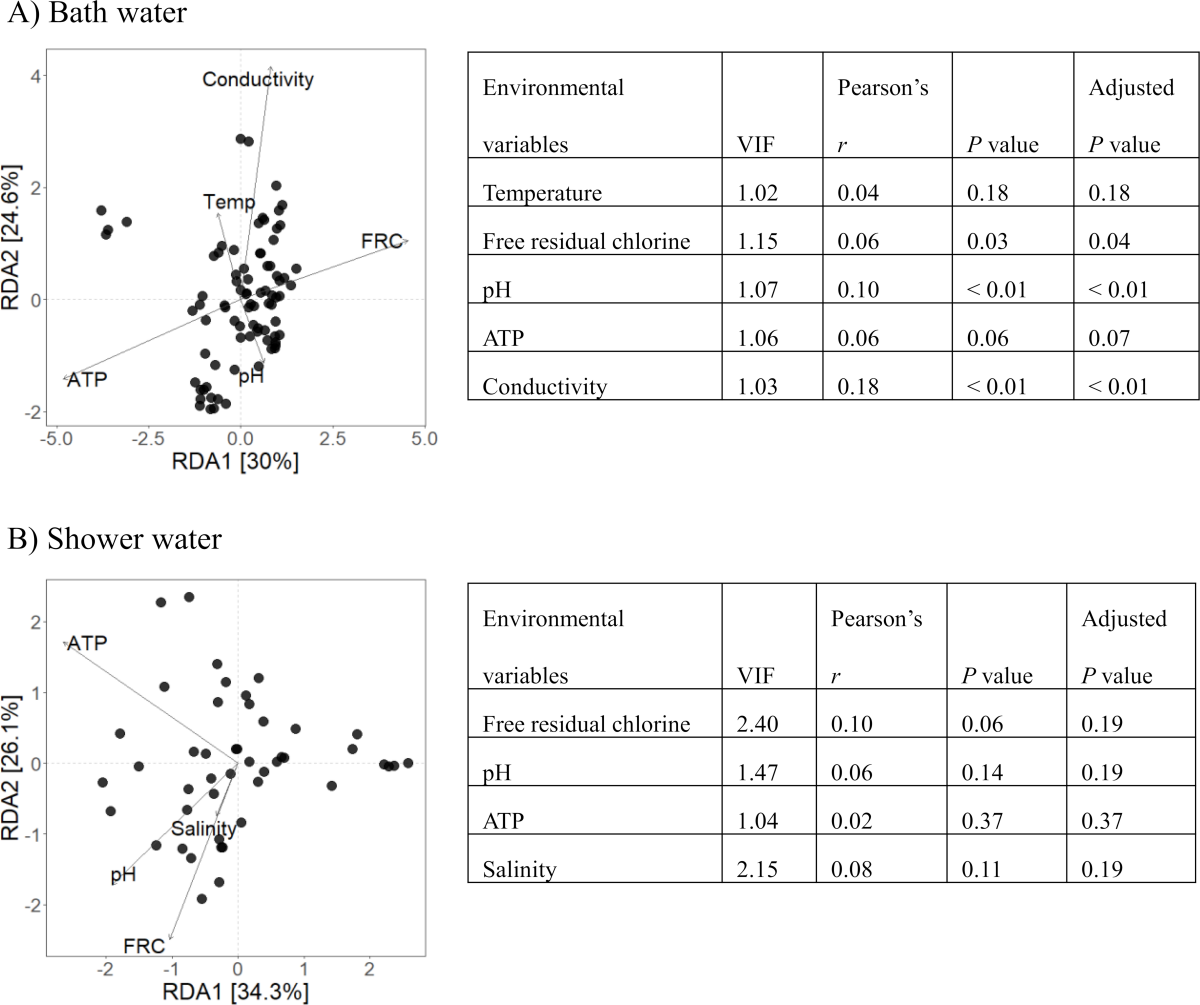
Bray-Curtis dbRDA showed the correlation between environmental variables and dissimilarity of bacterial community in bath water (A) and shower water (B). Bray-Curtis dbRDA was performed on the 128 samples (82 from bath water and 46 from shower water) for which environmental variables for all factors (temperature, free residual chlorine, pH, conductivity, total dissolved solids, salinity, and ATP) were obtained, by using Microeco package. Temperature was excluded in the analysis of shower water. We used Mantel test to check whether there were significant correlations between environmental variables and distance matrix. To avoid multicollinearity and misleading statistical results, the variables were excluded from the model if values of variance inflation factor (VIF) exceeded 10. Total dissolved solids and salinity were excluded in bath water. Conductivity and total dissolved solids were excluded in shower water.

The distributions of the water samples, including bath and shower water, are shown in the Bray-Curtis distance-based redundancy analysis (dbRDA) ordination plot (Fig. 5). In bath water, RDA1 and RDA2 explained 30.0% and 24.6% of the variation in the bacterial community, respectively. The results indicated that the bacterial community was significantly correlated with free residual chlorine, pH, and conductivity. In contrast, RDA1 and RDA2 explained 34.3% and 26.1% of the variation in the bacterial community in shower water, respectively, and the bacterial community was not significantly correlated with any environmental variables.

## DISCUSSION

In this study, we characterized the bacterial community present in the water of public bath facilities, along with their chemical parameters, and investigated the effect of the bacterial microbiome on the presence of *Legionella* species. Although it has not been investigated whether DNA from *Legionella* species detected in water samples was derived from viable but non-culturable (VBNC) or dead cells, multiple *Legionella* species may exist simultaneously in water samples. *L. pneumophila* is the most virulent species, causing the vast majority of *Legionella* infections. However, all species of the genus *Legionella* are potentially pathogenic in humans (32), indicating the importance of detection of non-*L*. *pneumophila* species identification. In this study, non-*L*. *pneumophila* species have rarely been isolated using the plate culture method. Our previous studies also showed that *L. pneumophila* was the most frequently detected strain using the plate culture method in water samples from baths, showers, and puddles (6, 33, 34). Thus, many non-*L*. *pneumophila* species may exist in the VBNC state in water from public bath facilities. Several stress factors induce *Legionella* cells to enter a VBNC state, but these cells can still directly infect human macrophages and amoebae, indicating that VBNC *Legionella* cells can cause diseases in humans (35, 36). Our 16S rRNA gene amplicon analysis indicates that plate culture methods may not accurately monitor *Legionella* in water. Jeong et al. reported that the short fragments of 16S rRNA gene amplicons (<500 bp in the present study) limit their use for 16S rRNA gene-based bacterial identification at the species level because of the sequence similarity of the variable region (37). Thus, amplicon analysis targeting other regions or genes is required for more detailed analyses at the species level, which will serve as a foundation for future research (38, 39). Further investigations using techniques, such as RNA-seq and reverse transcription-PCR sequencing, which can quantify viable cells (40), are required to obtain a detailed understanding of the state of bacteria, including *Legionella* species, in water samples.

LEfSe and SCNIC analyses suggest that *Legionella* species may interact directly or indirectly with many bacterial species. *Corynebacterium* was negatively correlated with *Legionella* species in the bath water samples. This result was consistent with the previous report that *L. pneumophila* was unable to attach to biofilms formed by *Corynebacterium* (41). *Tepidimonas* is known to be thermophilic bacterium often detected in hot springs (42). Therefore, in the samples where *Legionella* species, also known to be thermophilic bacterium (43), were present, *Tepidimonas* may also have been abundant. *Sphingomonas*, which was usually detected in engineered water systems (44), was negatively correlated with *Legionella* species in shower water samples. It has been reported that *Sphingomonas* appeared to have an antagonistic effect on the persistence of *L. pneumophila* (45, 46). Thus, our results were similar to those of previous studies. *Nitrospira* and *Leptospirillum*, members of the family Nitrospiraceae, were enriched in *Legionella*-positive samples. *Nitrospira*, aerobic nitrite-oxidizing bacterium, has been detected in biofilm and water from drinking water distribution system (47), especially containing elevated levels of iron and manganese (48). *Leptospirillum*, aerobic and acidophilic ferrous iron oxidizers, has been detected in biofilms of iron water pipes and contributed to iron pipe corrosion (49). These bacteria may interact with *Legionella* in the biofilm formed within shower water pipes. As shown in Figure 3B, many genera were positively correlated with *Legionella* in shower water. A possible explanation for this relationship is that these genera promote the growth or survival of *Legionella*. Alternatively, these genera, including *Legionella*, may be resistant to amoebal predation. Indeed, an increase in *Nitrospira* was observed after inoculation with amoebae in an experimental soil system (50). LEfSe and SCNIC analyses also suggest that *Legionella* species may interact directly or indirectly with several unassigned and uncultured bacteria. The present study reveals the ecology of *Legionella* species, especially their interactions with other bacteria that are poorly understood to date.

In bath water samples, *Pseudomonas* was the most frequently detected genus. This genus has also been detected in cooling towers and dental unit water samples (14, 15). Although Paranjape et al. reported a strong negative correlation between *Pseudomonas* and *Legionella* species, the same was not observed in this study. The richness and dissimilarity of the bacterial community were not affected by the water source (hot or non-hot spring water). However, several factors (free residual chlorine, pH, and conductivity) were correlated with the bacterial community in bath water. This may be explained by the fact that the characteristics of bath water are highly variable, whereas shower water tends to have similar characteristics owing to similar water treatment procedures. Other factors, such as the type of disinfectant (13), the normal microbiota of the users’ skin, and warm temperatures around 40 °C, common in bath water, may affect the bacterial community including *Legionella* species in bath water. *Mycobacterium* was detected as the higher-ranked bacterial genus in both bath and shower water samples. This suggests that public bath facilities may be a source leading to the increasing number of patients with nontuberculous mycobacterial diseases in recent years (51). In shower water samples, bacterial community richness and structure differed between samples collected from tap water and well water. Specifically, Alphaproteobacteria were highly abundant in samples derived from tap water. A previous study indicated that Alphaproteobacteria can tolerate more chlorination (52). This may be because taps are fed with treated municipal water in Japan, unlike hot springs or well water. *Phreatobacter* was the most frequently detected genus in shower water, as reported previously (53, 54). *Legionella* DNA was detected in more than half of the shower water samples, suggesting that the risk of *Legionella* inhalation should be considered, because showerheads easily generate aerosols during routine use. Although no significant difference in alpha diversity was observed between the bath and shower water samples, there was a remarkable difference in beta diversity. As indicated by the taxonomic composition, the dominant bacteria differed between bath water and shower water samples. LEfSe and SCNIC analyses also revealed that the bacterial species interacting with *Legionella* species differed between bath water and shower water. The alpha diversity of the bath water was low in samples containing low levels of free residual chlorine (<0.4 mg/L). These results indicate that a high degree of bacterial growth in water from public bath facilities reduces bacterial community richness and that specific bacterial species may be more likely to dominate.

In terms of hygiene management, it is important to reduce the growth of *Legionella* species by disinfecting the water from public bath facilities. Our findings contribute to the establishment of appropriate hygiene management practices and provide a basis for understanding the potential health effects of bath and shower water in public bath facilities. Since *Legionella* species are difficult to culture, our findings may serve as a basis for establishing a new method for monitoring *Legionella* species using bacteria that are highly correlated with *Legionella* species as indicators. Furthermore, we were interested in determining the sequence types of *L. pneumophila* isolates to examine species diversity and whether multiple *L. pneumophila* isolates coexist in these environments using the next-generation sequencing approach.

## MATERIALS AND METHODS

### Water samples

Nine public health centers (in the Niikawa, Uozu, Chubu, Toyama, Imizu, Takaoka, Himi, Tonami, and Oyabe districts) participated in the analysis of *Legionella* species prevalent in 27 public bath facilities in the Toyama Prefecture, Japan, between 2019 and 2021 (Table S1). Bath water (87 samples) and shower water (51 samples) were collected in sterile bottles (2 L each). Shower water was flushed out for approximately 10 s prior to sampling. The temperature (°C) and free residual chlorine concentrations (mg/L) were measured on-site by staff members of public health centers. Free residual chlorine concentrations were measured using the *N*,*N*-diethyl-*p*-phenylenediamine (DPD) method (55) via several commercial kits (Nippon Soda, Tokyo, Japan; Suzuken, Tokyo, Japan; Sibata Scientific Technology, Tokyo, Japan; and Wako Pure Chemical Industries, Osaka, Japan). The conductivity (mS) (56), total dissolved solids (mg/L) (57), and salinity (%) (58) were measured using a meter for multiple-purpose operations (CD-4307SD, Mother Tool, Nagano, Japan).

### Plate culture method

The plate culture method was performed in accordance with ISO 11731:2017 (59). Water samples were concentrated 100-fold by filtration through a 0.22-μm-pore-size polycarbonate membrane (Millipore, Billerica, MA, USA). The concentrated samples were divided into three portions (0.1 mL each) and cultured without pretreatment and after heat (50°C for 20 min) and acid treatment (equal volumes of 0.2 mol/L KCl-HCl buffer [pH 2.2] for 5 min at room temperature) on glycine-vancomycin-polymyxin B cycloheximide agar plates (Nikken Bio Medical Laboratory, Kyoto, Japan). Non-concentrated water samples were then plated. The agar plates were incubated at 35°C for 7 d in a humidified chamber. Smooth gray colonies with characteristic outward structures (cut-glass-like or mosaic-like appearance) viewed under a stereomicroscope with oblique illumination (60) were subcultured on buffered charcoal-yeast extract agar plates containing L-cysteine (bioMérieux, Lyon, France) and blood agar plates (Eiken Chemical, Tokyo, Japan). Colonies growing only on the buffered charcoal-yeast extract agar plates were presumed to belong to the genus *Legionella*.

Suspected colonies were confirmed to be *Legionella* species by quantitative polymerase chain reaction (qPCR) using the Cycleave PCR *Legionella* (16S rRNA) Detection Kit (Takara Bio, Shiga, Japan). The qPCR assay was positive for 74 *Legionella* strains (48 *Legionella* species) and negative for 14 non-*Legionella* strains (11 non-*Legionella* species: *Shigella sonnei*, *Escherichia coli*, *Vibrio parahaemolyticus*, *Campylobacter jejuni*, *Salmonella enterica*, *Clostridium botulinum*, *Clostridium perfringens*, *Staphylococcus aureus*, *Yersinia enterocolitica*, *Listeria monocytogenes*, and *Bacillus cereus*). *L. pneumophila* was identified by PCR with primers for *L. pneumophila* species-specific *mip* genes (61). Serogroups of *L. pneumophila* isolates were determined using a latex agglutination test kit (Oxoid, Hampshire, UK), and slide agglutination was performed with commercial antisera (Denka Seiken, Tokyo, Japan). The molecular characterization of *L. pneumophila* serogroup 1 isolates was performed according to the protocol of the European Society of Clinical Microbiology and Infectious Diseases Study Group for *Legionella* Infections sequence-based typing scheme (62, 63). Species of non-*L*. *pneumophila* were determined by sequencing the 16S rRNA genes with a 3500*xl* genetic analyzer (Thermo Fisher Scientific, MA, USA) and primers 27f (AGAGTTTGATCCTGGCTCAG) and 1492r (GGCTACCTTGTTACGACT T), as described previously (19). The detection limit was 10 CFU/100 mL.

### Next-generation sequencing for 16S rRNA gene amplicon

Water samples (1.2 L) were concentrated via filtration. For mechanical cell disruption, zirconia beads were added to the sample tube containing the filter and vortexed for 1 min. DNA was extracted using the DNeasy PowerBiofilm Kit according to the manufacturer’s instructions (Qiagen). A 16S rRNA gene amplicon library was prepared using the 16S Metagenomic Sequencing Library Preparation protocol for Illumina MiSeq (Illumina, San Diego, CA, USA). The bacterial 16S V3–V4 region was amplified by PCR using the Tks Gflex DNA Polymerase (Takara Bio) with the 16S Amplicon PCR Forward Primer (5′-TCGTCGGCAGCGTCAGATGTGTATAAGAGACAGCCTACGGGNGGCWGCAG-3′) and the 16S Amplicon PCR Reverse Primer (5′-GTCTCGTGGGCTCGGAGATGTGTATAAGAGACAGGACTACHVGGGTATCTAATCC-3′) and then sequenced using an Illumina MiSeq instrument with the MiSeq Reagent Kit v3 according to the manufacturer’s instructions (600 cycles).

### Bioinformatic processing

Bioinformatics analysis of the microbiome was performed using QIIME2 2021.4 (64). Raw sequence data were demultiplexed and quality-filtered using the q2-demux plugin, followed by denoising with DADA2 (65) (via q2-dada2). All ASVs were aligned with mafft (via q2-alignment) (66), which was used to construct a phylogeny using FastTree 2 (via q2-phylogeny) (67). Alpha and beta diversity metrics were estimated using q2-diversity after the samples were rarefied (subsampled without replacement) to 9,892 sequences per sample. Alpha diversity was calculated to quantify bacterial community richness. The Shannon diversity index was used to measure alpha diversity (68). Rarefaction curves indicated sufficient sequencing coverage, as demonstrated by the observation that the Shannon index accumulation curves reached a plateau at a sampling depth of 9,892 (Fig. S3). To quantify the dissimilarity of the bacterial communities, beta diversity was calculated using the Bray-Curtis distance (69). These metrics were used to assess the microbial communities in the water samples. The Bray-Curtis distance between the samples was visualized with an NMDS plot using the R statistical software package (version 4.1.2).

Taxonomy, from kingdom to species, was assigned to the ASVs using the q2-feature-classifier (70), based on the classify-sklearn naïve Bayes taxonomy classifier against the Silva v138 99% OTU database. Several taxa were not validly published in the LPSN. LEfSe analysis was performed to determine the significantly different taxa between *Legionella*-read positive and negative samples. The LDA scores indicated the degree of consistent differences in relative abundance between *Legionella-*positive and -negative samples. Different taxa with significant differences between sampling source types were assessed using the LEfSe program with the following parameters (alpha value for the pairwise Wilcoxon test set at 0.05; logarithmic LDA score threshold set at 3.0) using the Galaxy Hutlab online platform (71) (https://huttenhower.sph.harvard.edu/galaxy/). The modules in this study were defined as previously described (72). Modules are groups in which the organisms are strongly correlated and connected by an edge. To detect the modules, the q2-SCNIC plugin was used with default parameters. This method removed all ASVs with a read abundance below 500 and all ASVs with an average abundance of less than 2 across all samples. To calculate all the pairwise correlations between ASVs, the sparCC metric was used to measure the strength of the correlations. The minimum R value of 0.35 was set as the cutoff value to define the modules. SCNIC was used to summarize the modules and obtain a feature table that included all ASVs in the modules. Networks were visualized using Cytoscape and modified using Microsoft PowerPoint (Microsoft, Tokyo, Japan). To reveal the relationship between environmental variables and the dissimilarity of bacterial communities, Bray-Curtis dbRDA was performed on 128 samples (82 from bath water and 46 from shower water), for which environmental variables for all factors (temperature, free residual chlorine, pH, conductivity, total dissolved solids, salinity, and ATP) were obtained using the Microeco package (73). Temperature was excluded from the shower water analysis. We used the Mantel test to determine whether there were significant correlations between the environmental variables and the distance matrix. To avoid multicollinearity and misleading statistical results, variables were excluded from the model if values of variance inflation factor (VIF) exceeded 10. Total dissolved solids and salinity were excluded in bath water. Conductivity and total dissolved solids were excluded in shower water.

### Statistical analysis

The Shannon indices of the water samples were normally distributed according to the Shapiro–Wilk test (*P* > 0.05). After the F-test for the equality of variances, the Student’s *t*-test (equal variances) or Welch’s *t*-test (unequal variances) was applied to investigate the differences in the alpha diversity of the water samples. All tests were performed using Excel (Microsoft, Redmond, WA, USA) or the R statistical software package (version 4.1.2). *P* value significance was set at *P* < 0.05.

## Data Availability

The data sets supporting the conclusions of this article have been deposited in NCBI/ENA/DDBJ under accession numbers DRA016474 for the 16S rRNA gene amplicons from metagenomic DNA extracted from the water samples and LC789027 to LC789029 for the 16S rRNA genes of the isolates.

## SUPPLEMENTAL MATERIAL

Fig. S1 spectrum.03459-23-s0001.pdf Alpha diversity categorized by free residual chlorine.

Fig. S2 spectrum.03459-23-s0002.pdf Taxonomic composition at the class level.

Fig. S3 spectrum.03459-23-s0003.pdf Rarefaction curve for each sample.

Table S1 spectrum.03459-23-s0004.xlsx Sample information.

Table S2 spectrum.03459-23-s0005.xlsx Number of species for the genus *Legionella* in each sample.

Table S3 spectrum.03459-23-s0006.xlsx SCNIC analysis in bath water.

Table S4 spectrum.03459-23-s0007.xlsx SCNIC analysis in shower water.

ASM does not own the copyrights to Supplemental Material that may be linked to, or accessed through, an article. The authors have granted ASM a non-exclusive, world-wide license to publish the Supplemental Material files. Please contact the corresponding author directly for reuse.
