# Supplementary material for: Correlation between bacterial microbiome and Legionella species in water from public bath facilities by 16S rRNA gene amplicon sequencing
Source: Microbiol Spectr. 2024 Feb 16;12(4):e03459-23. doi: 10.1128/spectrum.03459-23 (PMC10986325; doi:10.1128/spectrum.03459-23)
Supplement: Fig. S1 — Alpha diversity categorized by free residual chlorine. [file spectrum.03459-23-s0001.pdf]

Supplementary Figure S1

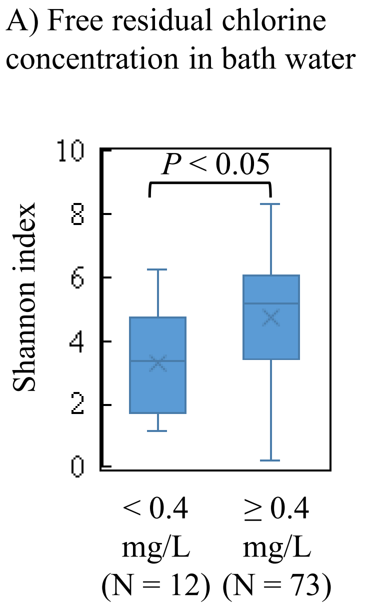

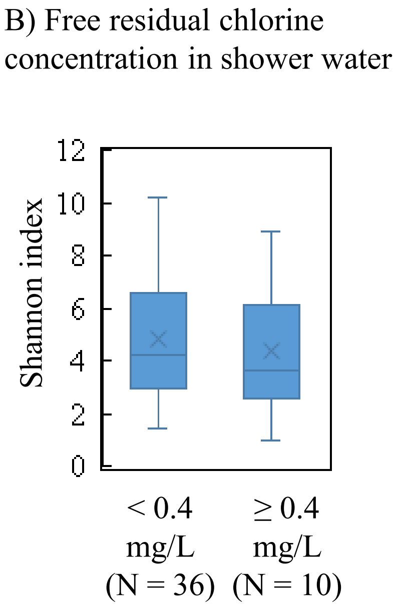

Supplementary Figure S1. Alpha diversity of water samples categorized by free residual chlorine concentration of bath water (A) and free residual chlorine concentration of shower water (B).
