# Supplementary material for: Correlation between bacterial microbiome and Legionella species in water from public bath facilities by 16S rRNA gene amplicon sequencing
Source: Microbiol Spectr. 2024 Feb 16;12(4):e03459-23. doi: 10.1128/spectrum.03459-23 (PMC10986325; doi:10.1128/spectrum.03459-23)
Supplement: Fig. S2 — Taxonomic composition at the class level. [file spectrum.03459-23-s0002.pdf]

Supplementary Figure S2

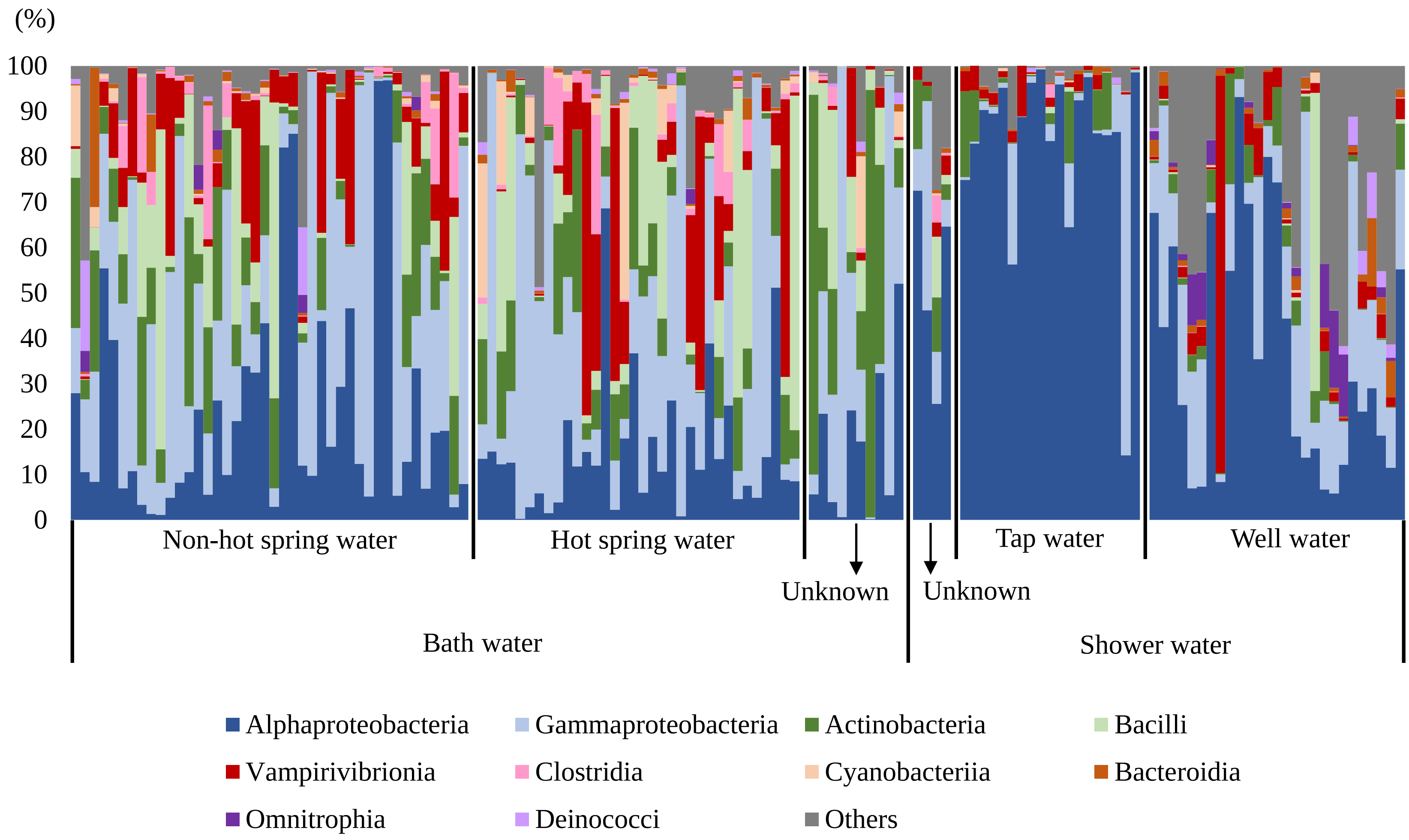

Supplementary Figure S2. Taxonomic composition of bacterial reads at the class level. Vampirivibrionia and Omnitrophia are not validly published class in the LPSN.
