# Supplementary material for: Correlation between bacterial microbiome and Legionella species in water from public bath facilities by 16S rRNA gene amplicon sequencing
Source: Microbiol Spectr. 2024 Feb 16;12(4):e03459-23. doi: 10.1128/spectrum.03459-23 (PMC10986325; doi:10.1128/spectrum.03459-23)
Supplement: Fig. S3 — Rarefaction curve for each sample. [file spectrum.03459-23-s0003.pdf]

Supplementary Figure S3

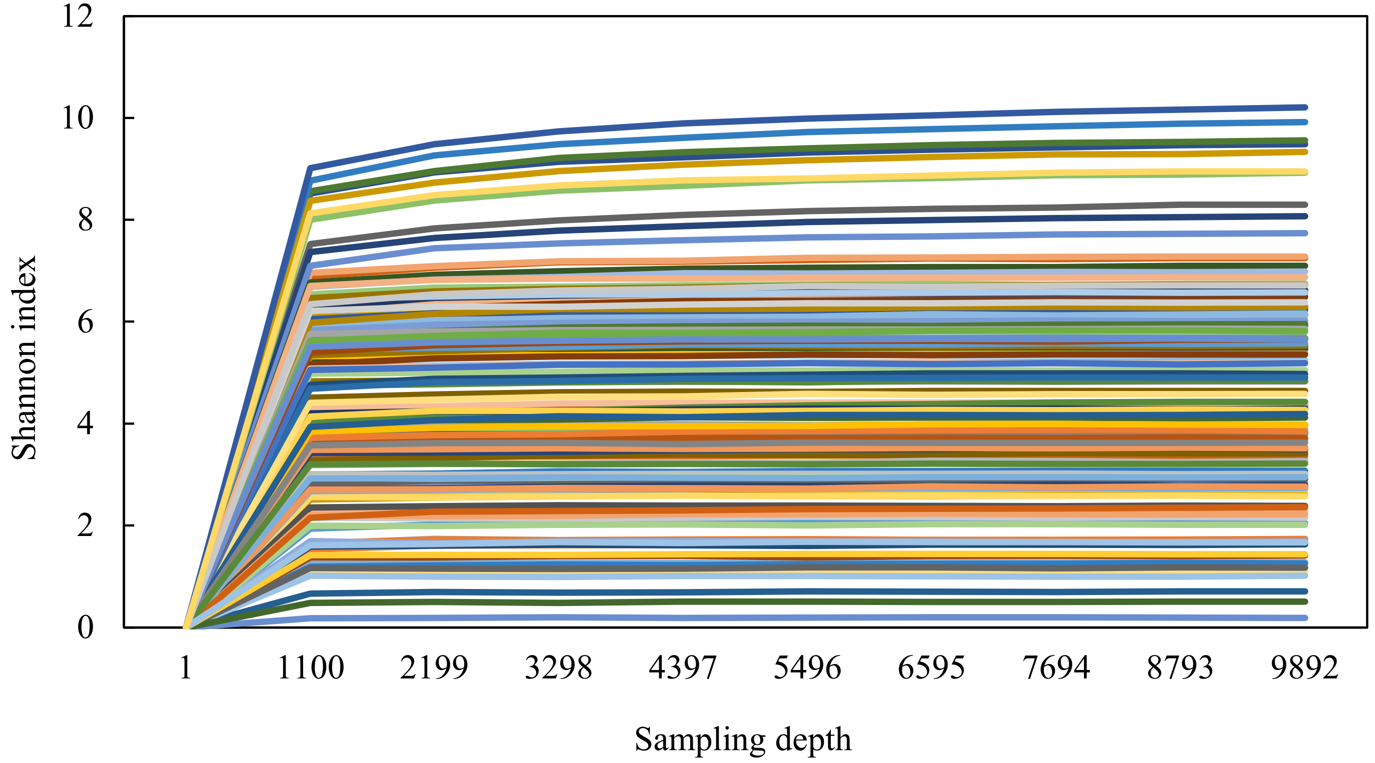

Supplementary Figure S3. Rarefaction curve for each sample based on the Shannon index.
